# Feature extraction of static foam images based on U-Net and Hough transform

**DOI:** 10.1371/journal.pone.0362662

**Published:** 2026-11-11

**Authors:** Deqiang Peng, Daoguang Li, Qingxia Li, Chenyi Wang, Xiaomin Ma

**Affiliations:** 1 College of Energy and Mining, Heilongjiang University of Science and Technology, Harbin, China; 2 Department of Mineral Processing Engineering, Taiyuan University of Technology, Taiyuan, China; Kohat University of Science and Technology (KUST), PAKISTAN

## Abstract

In flotation-based mineral processing, froth images contain important visual information that reflects the operating condition of the process and provides a basis for process monitoring and control. However, conventional machine-vision methods still encounter difficulties in extracting static froth features under conditions of bubble adhesion, overlap, weak boundaries, and uneven illumination. To address these problems, a flotation froth image analysis framework is proposed by combining a boundary-aware U-Net segmentation model with a segmentation-guided adaptive Hough transform. In the first stage, the U-Net architecture is enhanced with explicit boundary-oriented supervision to improve the separation of adherent and overlapping bubbles. In the second stage, the geometric priors derived from the segmentation results are used to constrain the Hough-based detection process, thereby improving the reliability of bubble counting and geometric feature extraction. Experimental results show that the proposed method achieves a *Dice* similarity coefficient of 96.39% and an intersection over union of 93.04%, while maintaining favorable inference efficiency. Compared with representative segmentation models, the proposed framework provides a better balance between accuracy and speed. The study demonstrates that the integration of deep boundary-aware segmentation and adaptive geometric detection is effective for static feature extraction of flotation froth images and has potential for intelligent monitoring of flotation processes.

## 1. Introduction

Froth flotation is one of the most important unit operations in mineral processing, in which selective separation is achieved based on the differences in the surface hydrophobicity of minerals [1]. In industrial production, foam is a common phenomenon in a wide range of processes, including mineral separation, biological fermentation, chemical reactions, and water treatment. Among these applications, the role of foam is particularly critical in mineral flotation processes.

In coal preparation plants, the increasing degree of mechanization has led to a gradual rise in the proportion of fine coal particles, which has consequently increased the pressure on flotation operations. As a result, the performance of coal flotation has become a key factor restricting the sustainable development of coal preparation plants. Therefore, achieving economical and efficient separation of fine coal has become one of the primary challenges currently faced by coal processing facilities [2].

Since the flotation process mainly involves the interaction between air bubbles and fine particles during mineralization, the characteristics of bubbles on the froth surface—such as bubble size, color, and texture—can directly reflect the operational status of the flotation system, the quality of the products, and the efficiency of resource utilization. In recent years, numerous researchers both domestically and internationally have carried out extensive studies on the optimization and innovation of coal processing and mineral beneficiation technologies [3].

In the traditional coal preparation industry, the control of the flotation process still mainly relies on manual visual monitoring. This traditional method faces two serious problems: 1) Human vision is prone to fatigue, making it difficult to maintain continuous observation of the foam morphology characteristics within the flotation tank, directly leading to delays in the adjustment of process parameters, which in turn causes fluctuations in production efficiency. 2) Operators with different technical backgrounds have significant discrepancies in their understanding of foam conditions. Experienced operators often rely on their experience to estimate product quality based on foam status to adjust the flotation state, while less experienced staff tend to follow operational procedures for flotation. This cognitive difference directly translates into a discrete distribution of control strategies, resulting in inconsistent fluctuations in production process parameters over time.

These technical bottlenecks collectively point to the essential requirement for controlling the flotation process—establishing a machine vision-based quantitative analysis system for foam states. This technological transformation can not only enhance the stability and economics of production processes but also serves as a concrete practice for advancing traditional mining towards digitalization and intelligent upgrades.

Traditional approaches for extracting static features from froth images mainly fall into three categories: edge detection, watershed-based segmentation, and threshold segmentation. Conventional edge operators, such as the Sobel and Roberts operators, are generally capable of identifying only the bright regions in the central parts of bubbles, whereas the edge information of the froth is often lost to a considerable extent [4,5]. To alleviate the over-segmentation problem inherent in the watershed method, Gong J [6] proposed a fuzzy clustering scheme for post-processing watershed results, thereby obtaining multilevel image segmentation.Zhang W et al. [7] developed a froth image processing algorithm in Python that integrates watershed segmentation with morphological operations, which improves both the accuracy of bubble delineation and the efficiency of bubble-size measurement in flotation froths. Saraji W. et al. [8] presented a hybrid semantic segmentation method combining U-Net and the watershed algorithm, where U-Net is used to extract froth-region features and watershed processing is further applied to refine bubble boundaries, leading to more accurate segmentation of flotation froth images. Liang XM. et al. [9] proposed a segmentation method based on highlight correction and adaptive parameter adjustment, which reduces the adverse influence of specular reflection and illumination variation on segmentation results and improves the accuracy of bubble-boundary recognition. Lu M [10], by combining grayscale conversion, histogram equalization, and threshold segmentation, achieved effective extraction of bubble counts. Nevertheless, under conditions of complex froth structure and low image contrast, these traditional methods still suffer from indistinct boundaries and insufficient adaptability.

In recent years, U-Net and its variants have been widely employed in image processing. In the field of industrial material inspection, Chai J et al [11–13], focusing on different scenarios such as gangue slurry, fabric defects, and steel-surface defects, introduced residual connections and depthwise separable convolutions to construct improved Res-UNet models that are both lightweight and highly accurate; these models significantly reduced computational cost while preserving segmentation performance. In addition, Hwang JW. et al. [14] reported an online inspection system for heating films based on U-Net, which achieved a regional detection accuracy of 96.6% and a defect detection rate of 97.5% at a conveyor speed of 12 m/min. U-Net has also produced notable results in medical imaging. Zhou L and Zhu X [15] applied U-Net to lung-tumor segmentation in CT images and obtained efficient segmentation performance.Hu Y et al. [16] further improved the recognition accuracy of liver-tumor boundaries by proposing a cascaded DDR-UNet++ framework. The successful application of U-Net across multiple fields has provided useful ideas for its introduction into mineral flotation studies.

At present, bubble-number extraction from static froth features is mainly based on thresholding combined with watershed segmentation, Mask R-CNN, and density-map regression. Although threshold segmentation and watershed algorithms are widely used, their performance remains unsatisfactory when dealing with severe bubble overlap and adhesion. By comparison, Hough-transform-based detection methods, owing to their sensitivity to geometric shape and strong robustness, have demonstrated high accuracy in specific applications, and a number of improved variants have been developed to accommodate more complicated bubble morphologies. Miao S et al. [17] first removed interference information from the image, then used an eight-point method to synchronously determine circle centers and radii, and finally performed candidate-circle re-search and optimal-circle selection to achieve accurate detection of circular targets. This method improved both the accuracy and efficiency of circle detection and exhibited stronger resistance to noise and disturbance. Wang H et al. [18] employed an improved Hough transform to identify the effective contour region and then combined curvature entropy with the least-squares method for curve fitting, thereby achieving complete contour extraction. Zhang X et al. [19] proposed an improved contrast-limited adaptive histogram equalization method for image enhancement and introduced a multiscale Hough detection algorithm to realize effective detection of pellets, thus improving production efficiency, reducing labor cost, and increasing the qualification rate of pellet preparation. In addition, Yao Z et al. [20] enhanced the Hough transform by incorporating curvature information of edge points, which significantly improved the efficiency of circle detection while maintaining detection accuracy. These studies indicate that, through morphological adaptation and algorithmic enhancement, the Hough transform can provide more reliable extraction of froth features. Although the conventional Hough circle detector is most suitable for cases where bubbles are nearly circular and free of adhesion, sufficiently accurate detection can be achieved once U-Net has first produced a high-quality segmentation result. Therefore, the combination of U-Net-based segmentation and Hough circle detection has considerable research value.

Although U-Net has achieved remarkable success in medical and industrial image segmentation, its application to flotation froth analysis is still at an early stage. As early as the 1990s, machine vision had already been used to monitor flotation processes and extract morphological features such as froth size [21]. However, owing to the limitations of the imaging technology and algorithms available at that time, the segmentation accuracy for heavily overlapped and adhered bubbles was still far from satisfactory. In recent years, in order to make better use of machine vision in flotation, a number of improvements have been introduced into froth image recognition and segmentation algorithms. Tang Z et al. [22] applied an Inception-based structure together with a self-attention mechanism to the segmentation of zinc-flotation froth images, thereby alleviating both under-segmentation and over-segmentation. Fan Y. et al. [23], aiming at the common problems of boundary fracture and size deviation in flotation froth segmentation, proposed a parallel-branch network with hybrid-loss supervision, which preserves high-resolution features more effectively and improves bubble-boundary quality. Zhou X and He Y [24] constructed a deep ensemble recognition sensor based on pretrained models, K-fold training, image augmentation, and TOPSIS-based ensemble decision-making, significantly improving the generalization ability and robustness of flotation froth operating-condition recognition. Although these methods have enhanced bubble-boundary identification to some extent, their potential for multiscale feature fusion and static-feature extraction has not yet been fully exploited.

Even though U-Net and its variants have proved effective in many image segmentation tasks, their application to flotation froth images must still address several specific challenges. Severe adhesion and overlap between bubbles require the model to possess a strong ability to discriminate adjacent edges. At the same time, the local high-contrast pattern formed by the bright spot in the bubble center and the darker surrounding boundary requires the network to capture this distinctive texture reliably. The classical U-Net, with its symmetric encoder-decoder architecture and skip connections, provides a good balance between the preservation of fine details and the fusion of high-level semantic information. It has shown particular strength in handling biomedical images with complex boundaries, a characteristic that is intrinsically consistent with the segmentation demands of froth images. For this reason, the classical U-Net was selected as the backbone network for the present segmentation task and serves as the foundation of the proposed two-stage analysis framework. The main contribution of this work does not lie in the simple application of U-Net itself, but rather in the construction of a task-oriented collaborative framework that deeply integrates deep learning with traditional geometric analysis [25]. In this framework, U-Net is employed to solve the problem of difficult segmentation and to generate favorable input conditions for the subsequent Hough transform, so that accurate extraction of static froth features can be achieved through the joint action of the two methods.

In view of the above considerations, this study develops a task-oriented framework for static feature extraction from flotation froth images. Unlike conventional approaches that directly apply standard segmentation or circle-detection algorithms, the proposed method combines a boundary-aware U-Net with a segmentation-guided adaptive Hough transform. The segmentation stage is designed to enhance the discrimination of weak, adherent, and overlapping bubble boundaries, while the subsequent geometric detection stage makes use of the structural information provided by the segmentation output to improve the robustness of bubble counting and size estimation. From the viewpoint of engineering application, the framework is developed under the practical constraint of limited industrial image data and emphasizes a favorable trade-off between segmentation accuracy, computational efficiency, and deployment potential. Therefore, the present work aims not only to improve the extraction of static froth features, but also to provide a more reliable visual-analysis basis for future intelligent flotation monitoring and control.

## 2. Problem description

The flotation froth image dataset used in this study was obtained from laboratory coal flotation experiments. Images were captured from the central region of the flotation cell, as illustrated in Fig 1. This region is generally considered to be the most representative observation area of the froth layer because the bubbles have reached a relatively mature and stable state after the flotation process develops. Consequently, the froth morphology in this region can more accurately reflect the actual separation behavior of the flotation system and provides reliable visual information for process monitoring.

**Fig 1:**
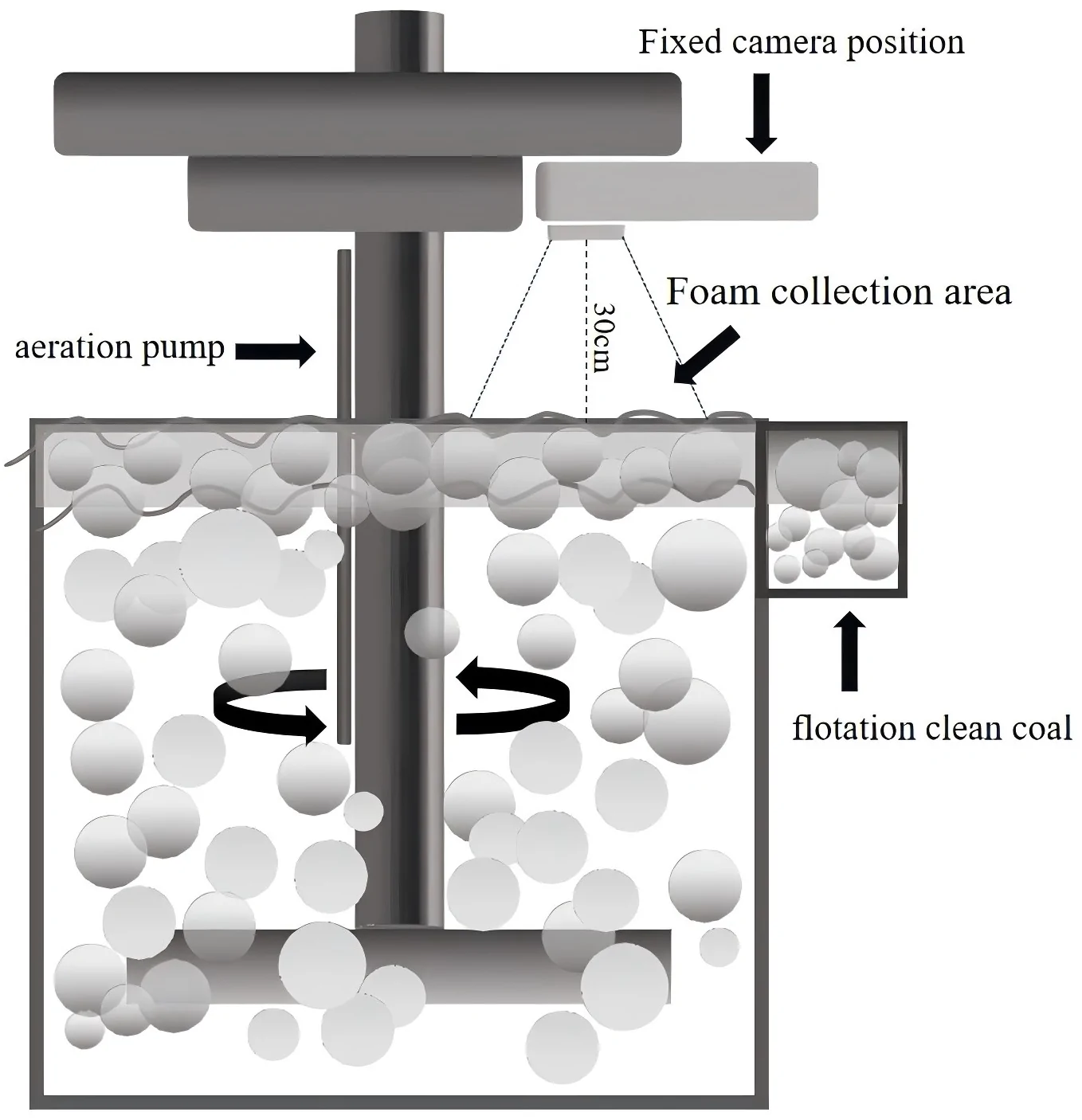
Schematic diagram of foam collection principle.

A high-speed industrial camera equipped with a 16 mm fixed-focus lens and an annular LED light source was used for image acquisition. The illumination color temperature was approximately 5500 K, and the camera was positioned about 300 mm above the froth surface in a perpendicular orientation.

A total of 500 images were initially collected, among which 300 images with clear froth structures were selected as valid samples. All images were resized to a unified resolution of 256 × 256 pixels to construct the froth image dataset.

The dataset comprises the original image (see Fig 2) and the bubble boundary annotation map (see Fig 3). The annotated image is a manually drawn contour map, which is used to guide the network to delineate the boundary of each bubble during the training process.

**Fig 2:**
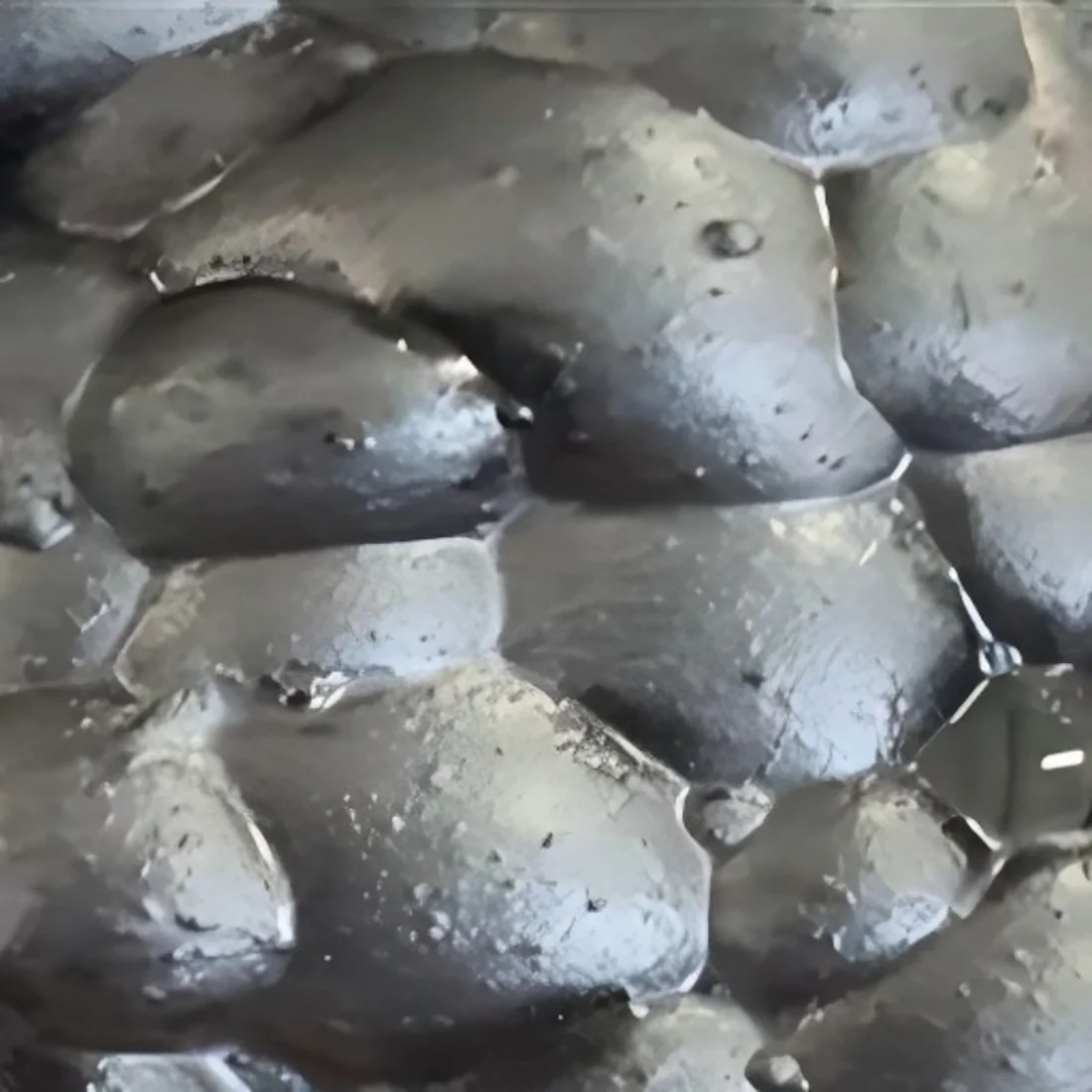
Original foam figure.

**Fig 3:**
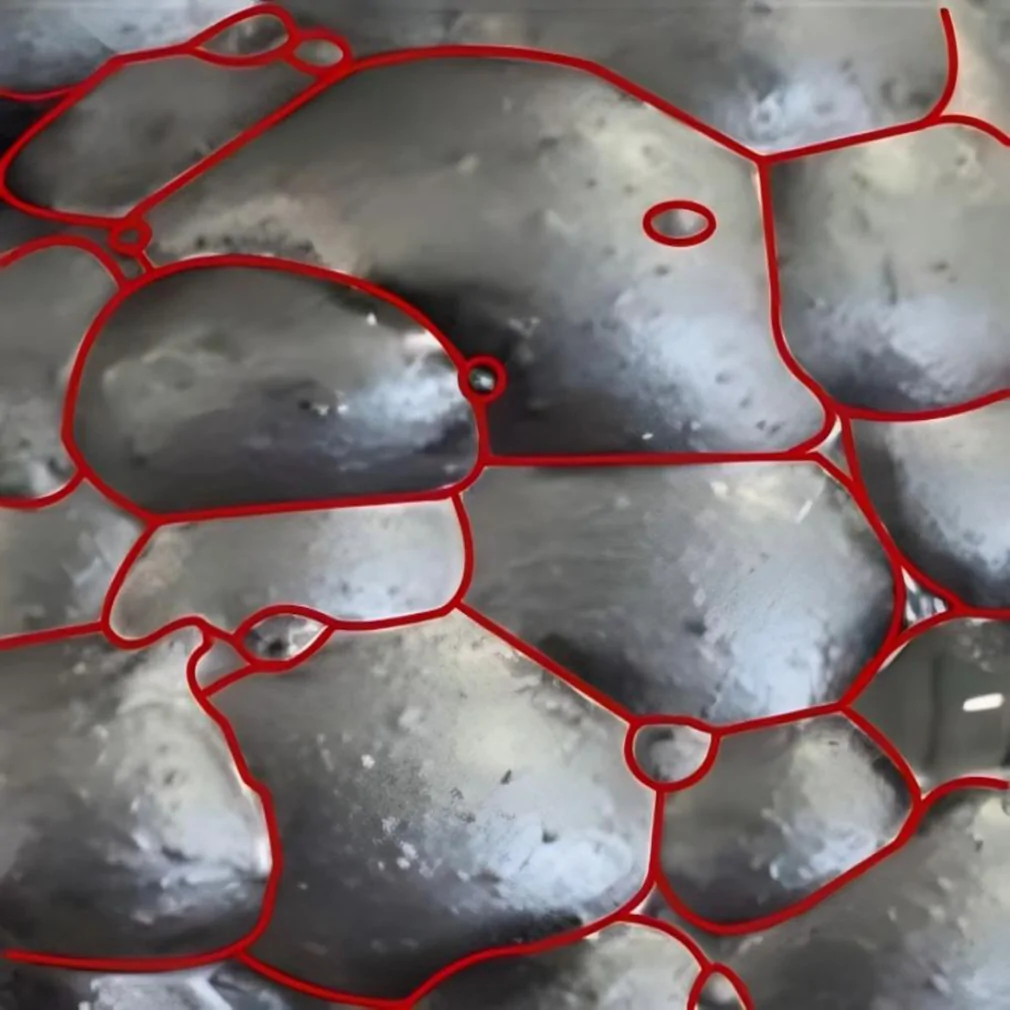
Foam edge marking figure.

The foam images captured during the flotation process mainly contain apparent visual information of the foam layer, and foams of different morphologies exhibit distinct static characteristics. Fig 4 illustrates representative foam morphologies and their corresponding static characteristics observed in the same flotation cell under varying operational conditions.

**Fig 4:**
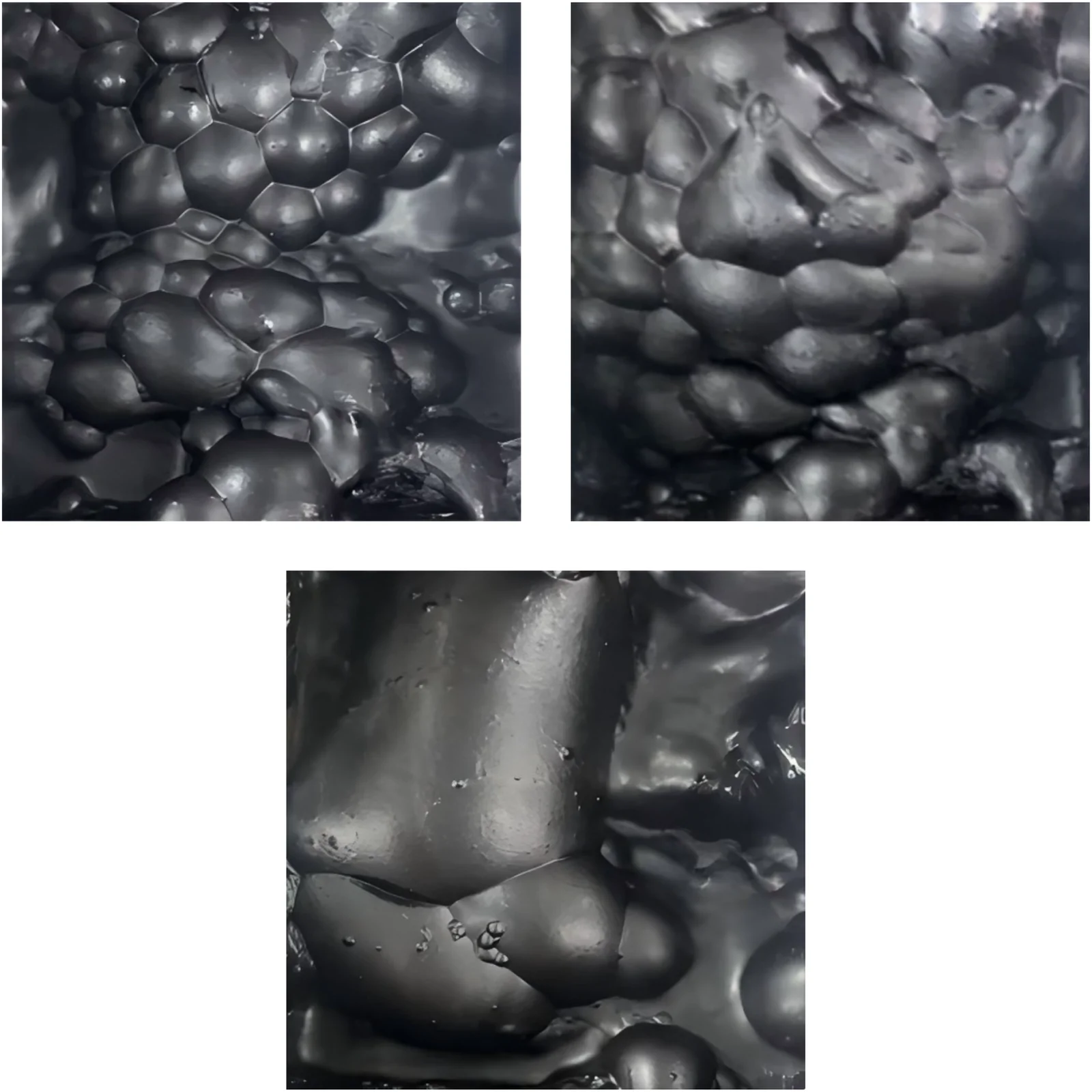
Static diagrams of foam in different forms.

We observed through extensive analysis of bubble image features that they exhibit the following characteristics: severe bubble adhesion; uneven bubble size; specular highlights at bubble centers; and low contrast.

To address the aforementioned challenges, Liu X. and Aldrich C [26]. employed semantic segmentation techniques [27,28] to achieve high-accuracy recognition of distinct flotation froth operating conditions. This methodology constitutes a significant advancement in image-based feature extraction, enabling dense, pixel-level classification. In this work, we adopt the U-Net architecture exclusively for semantic segmentation—leveraging its encoder-decoder structure and skip connections to preserve spatial fidelity while integrating contextual semantics. Relative to conventional segmentation approaches, U-Net demonstrates superior generalization capability, yielding enhanced segmentation accuracy and more precise boundary delineation. Building upon this foundation, we propose a deep learning–driven segmentation strategy to robustly extract froth regions and their morphological boundaries. The resulting segmentation masks serve as structural priors to guide subsequent geometric analysis, thereby establishing a two-stage quantitative framework for froth image interpretation.

## 3. Model Structure

The first stage of the proposed framework performs semantic segmentation of froth images using a U-Net-based architecture. The network follows a symmetric encoder–decoder structure designed to capture multi-scale visual features while preserving spatial information.

As illustrated in Fig 5, the encoder comprises four successive downsampling stages that hierarchically extract multi-scale feature representations from the input image. Each stage applies convolutional layers with 3 × 3 kernels and nonlinear activation functions (e.g., ReLU) to progressively abstract semantic content. With increasing network depth, the number of feature channels grows from 64 to 1024, while spatial resolution is halved at each downsampling step.

**Fig 5:**
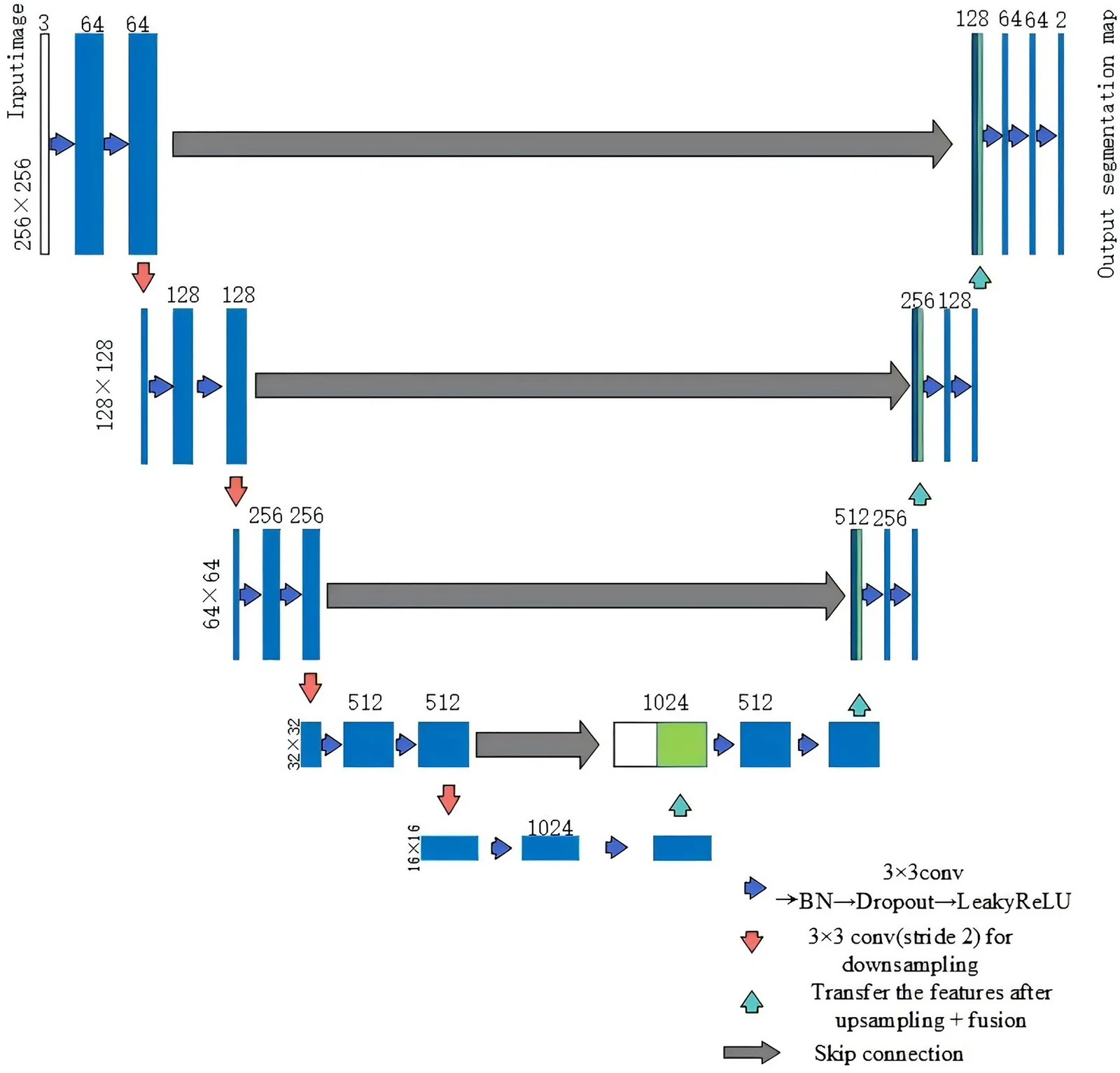
U-Net network structure.

The decoder path reconstructs high-resolution segmentation maps through progressive upsampling. Nearest-neighbor interpolation is employed for upsampling, followed by convolutional layers to refine contextual features. Crucially, skip connections bridge corresponding encoder and decoder levels, enabling fusion of low-level spatial details with high-level semantic representations. This architectural choice ensures faithful preservation of bubble boundary delineation and enhances segmentation accuracy.

These skip connections implement direct lateral pathways that concatenate encoder feature maps with upsampled decoder features at matching resolutions—thereby facilitating precise spatial localization and mitigating gradient degradation in deep supervision. The final layer projects 64-channel features to a single-channel output via a 3 × 3 convolution and applies a sigmoid activation to produce pixel-wise froth probability maps.

To support downstream geometric quantification, we integrate the U-Net segmentation model with the Hough circle transform, forming a unified two-stage analysis pipeline. This integration provides a robust foundation for extracting bubble size distributions, spatial density metrics, and other quantitative froth characteristics. The resulting framework achieves an optimal trade-off between computational efficiency and segmentation fidelity, demonstrating principled synergy between data-driven deep learning and geometry-aware classical computer vision.

### 3.1 Boundary sensing mechanism and parameters

To enhance the precision of boundary extraction in flotation froth images—particularly within ambiguous and weakly-defined regions—we empirically determine and fix the key parameters of the boundary extraction stage, as specified in Table 1.

**Table 1:** Settings for Post-processing Parameters of Boundary Map.

Processing Stage	Method	Parameter Settings
Input Image	Uniform scaling	256 × 256 px
Output Image Generation	U-Net + Sigmoid	Number of output channels = 3; Sigmoid [0,1]
Color Space Transformation	RGB to BGR conversion; HSV	OpenCV HSV color space
Red Boundary Extraction	HSV threshold segmentation	Lower bound: [0,70,120] Upper bound: [10,255,255]
Mask Generation	Cv2.inRange()	Boundary pixel value = 255; Background pixel value = 0
Contour Detectio	Cv2.findContours()	Cv2.RETR_EXTERNAL
Contour Compression	Contour approximation	Cv2.CHAIN_APPROX_SIMPLE
Coordinate Restoration	Reverse coordinate mapping	Scale; x_offset and y_offset
Result Overlay	Cv2.drawContours()	Red contours; line thickness = 4 px

The boundary extraction parameters specified in Table 1 were held fixed throughout the experiment and applied uniformly to all test images. This standardized parameterization ensured procedural consistency across samples and directly supported the reproducibility of segmentation outcomes by eliminating variability introduced through image-specific parameter tuning.

### 3.2 Data enhancement

In industrial machine vision applications, especially for challenging tasks such as flotation froth state analysis, acquiring large-scale, diverse, and high-quality annotated datasets remains a significant bottleneck due to high annotation costs and prolonged curation timelines.

To address this limitation, we apply geometric data augmentation to the training set, implementing five deterministic transformations: 90°, 180°, and 270° rotations, as well as horizontal and vertical flips, as illustrated in Fig 6.

**Fig 6:**
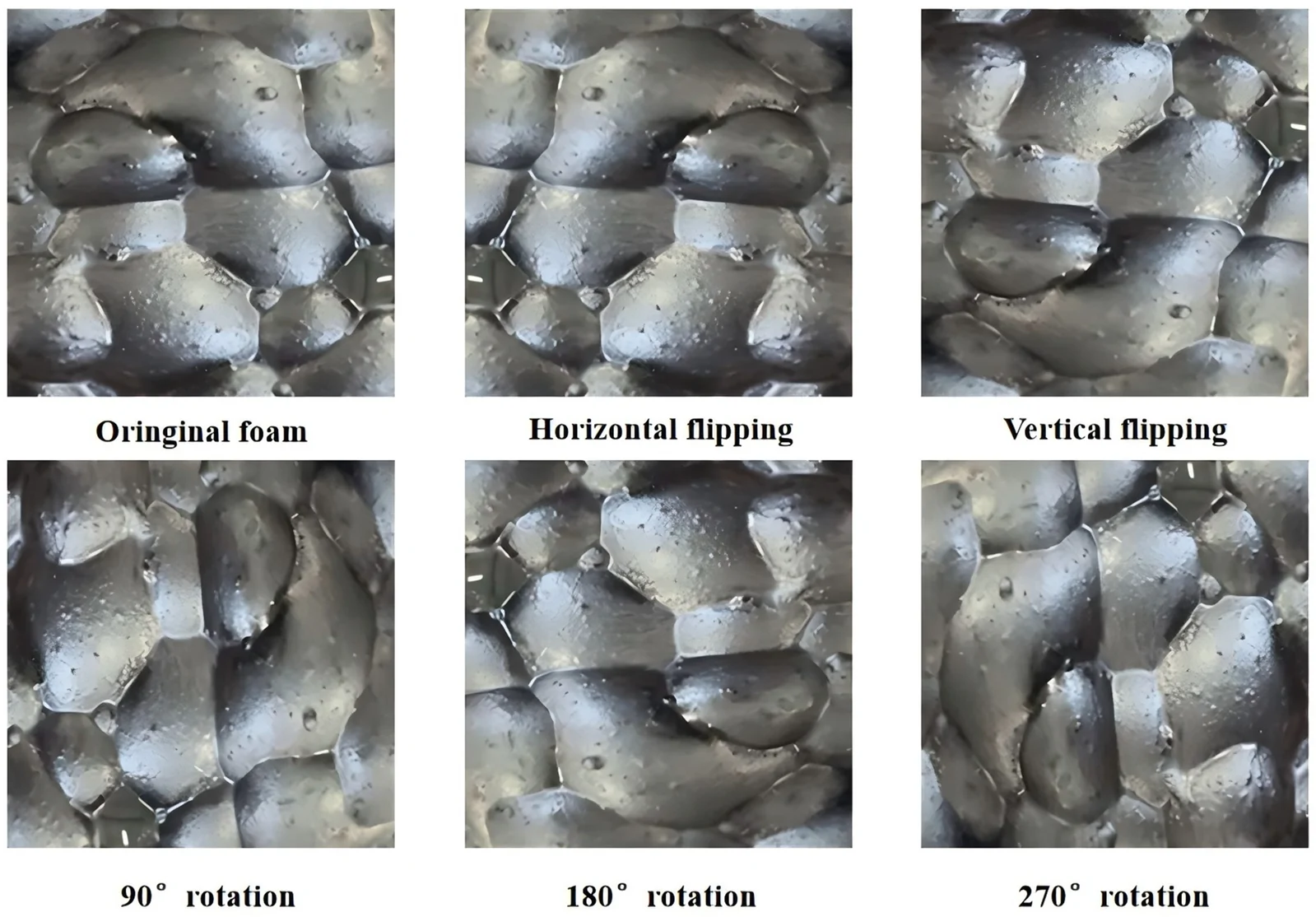
Original and augmented flotation foam images.

This data augmentation strategy mitigates data scarcity and simultaneously enhances the model’s generalization performance and robustness. Furthermore, the system integrates a configurable preprocessing pipeline supporting modular operations—including Gaussian denoising (with a 5 × 5 kernel), mean filtering, and Otsu binarization—which collectively improve image quality.

### 3.3 Loss function and optimizer

During model training, we employ the binary cross-entropy loss function (BCELoss) as the optimization objective. This loss computes stable and well-defined gradients by quantifying the pixel-wise divergence between predicted class probabilities and ground-truth labels. Its mathematical formulation is given by:


$$\begin{array}{c} L=-\frac{\text{1}}{N}\sum_{\text{1}}^{N}y_{i}log\left(y_{i}\right)+\left(\text{1}-\hat{y_{i}}\right)log\left(\text{1}-y_{i}\right) \end{array}$$ (1)

In this context, *N* is the total number of pixels,$y_{i}$ is the real label manually labeled (1 = Foam, 0 = Background), and $\hat{y_{i}}$ is the probability value predicted by the model.

In parallel, we employ the Adam optimizer to optimize model parameters, with update rules defined as follows:


$$\begin{array}{c} m_{t}=\beta_{\text{1}}m_{t-\text{1}}+\left(\text{1}-\beta_{\text{1}}\right)g_{t} \end{array}$$ (2)

$$\begin{array}{c} \nu_{t}=\beta_{\text{2}}\nu_{t-\text{1}}+\left(\text{1}-\beta_{\text{2}}\right)g_{t}^{\text{2}} \end{array}$$ (3)

$$\begin{array}{c} v_{t}=\beta_{\text{2}}v_{t-\text{1}}+\left(\text{1}-\beta_{\text{2}}\right)g_{t}^{\text{2}} \end{array}$$ (4)

$$\begin{array}{c} {\hat{m}}_{t}=\frac{m_{t}}{\text{1}-\beta_{\text{1}}^{t}} \end{array}$$ (5)

$$\begin{array}{c} {\hat{v}}_{t}=\frac{v_{t}}{\text{1}-\beta_{\text{2}}^{t}} \end{array}$$ (6)

$$\begin{array}{c} \theta_{t}=\theta_{t-\text{1}}-\alpha \frac{{\hat{m}}_{t}}{\sqrt{{\hat{v}}_{t}+\varepsilon }} \end{array}$$ (7)

In the formula, $m_{t}$、$v_{t}$: First-order and second-order moments;${\hat{m}}_{t}$、${\hat{v}}_{t}$: Bias-corrected first-order and second-order moment estimates; $\beta_{\text{1}}$, $\beta_{\text{2}}$: Momentum decay coefficients;$g_{t}$: Current parameter gradient; ε: Numerical stability constant (default: 1e-8); α: Learning rate (default: 0.001); $\theta_{t}$: Updated model parameters.

During training, the binary cross-entropy loss (BCELoss) guides parameter optimization by quantifying the pixel-wise divergence between predicted class probabilities and ground-truth labels—enabling the model to discriminate foam from background based on discriminative visual features. Concurrently, the Adam optimizer adaptively adjusts the learning rate: it employs larger update steps in the early training phase to accelerate convergence, and progressively reduces step sizes in later phases to stabilize optimization and mitigate gradient oscillation. This synergistic combination of BCELoss and Adam enhances both the convergence speed and segmentation accuracy of the model in flotation froth image segmentation.

### 3.4 Hough Transform Feature Detection Module

The core task of Hough transform is to detect circular bubbles from the segmentation results and calculate the attributes. The process is shown in Fig 7 below.

**Fig 7:**
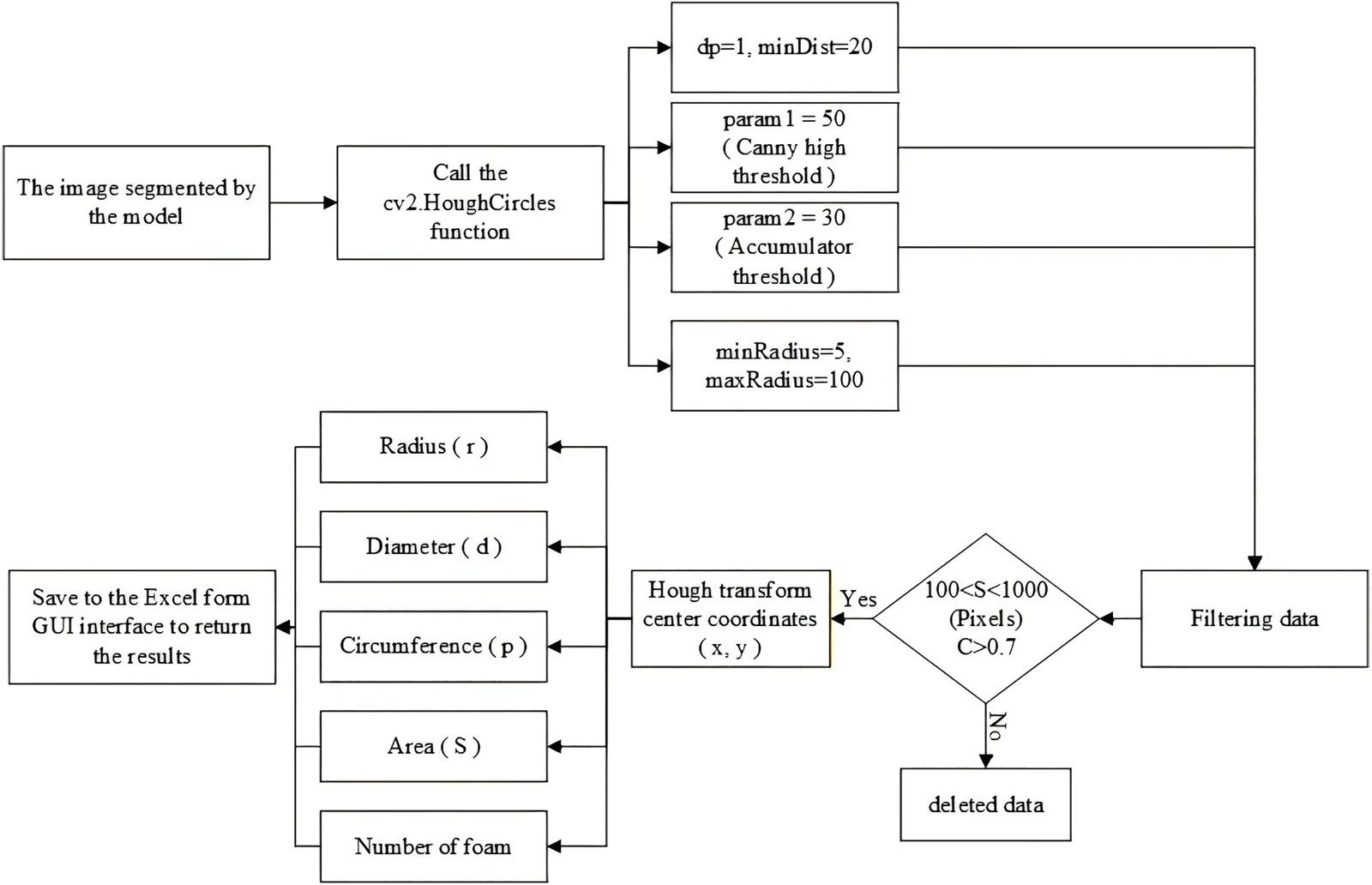
Hough Circle Detection Process.

Through this process, our method detects bubbles and estimates their count and geometric attributes—including center coordinates and radii—from the segmented image. We employ the Canny edge detector to extract edge pixels and compute their gradient directions. Subsequently, we invoke the “cv2.HoughCircles” function to construct an accumulator array in the three-dimensional parameter space (a, b, r), derived from the general equation of a circle: $(x-a{)}^{\text{2}}+(y-b{)}^{\text{2}}=r^{\text{2}}$.

Each edge pixel casts a vote for candidate circles in the parameter space (a, b, r) based on its gradient direction. Non-maximum suppression is then applied to localize prominent peaks in this space, which correspond to the most probable circle parameters. These detected parameter tuples (a, b, r) directly encode the center coordinates and radius of candidate circles in the input image, as illustrated in Fig 8.

**Fig 8:**
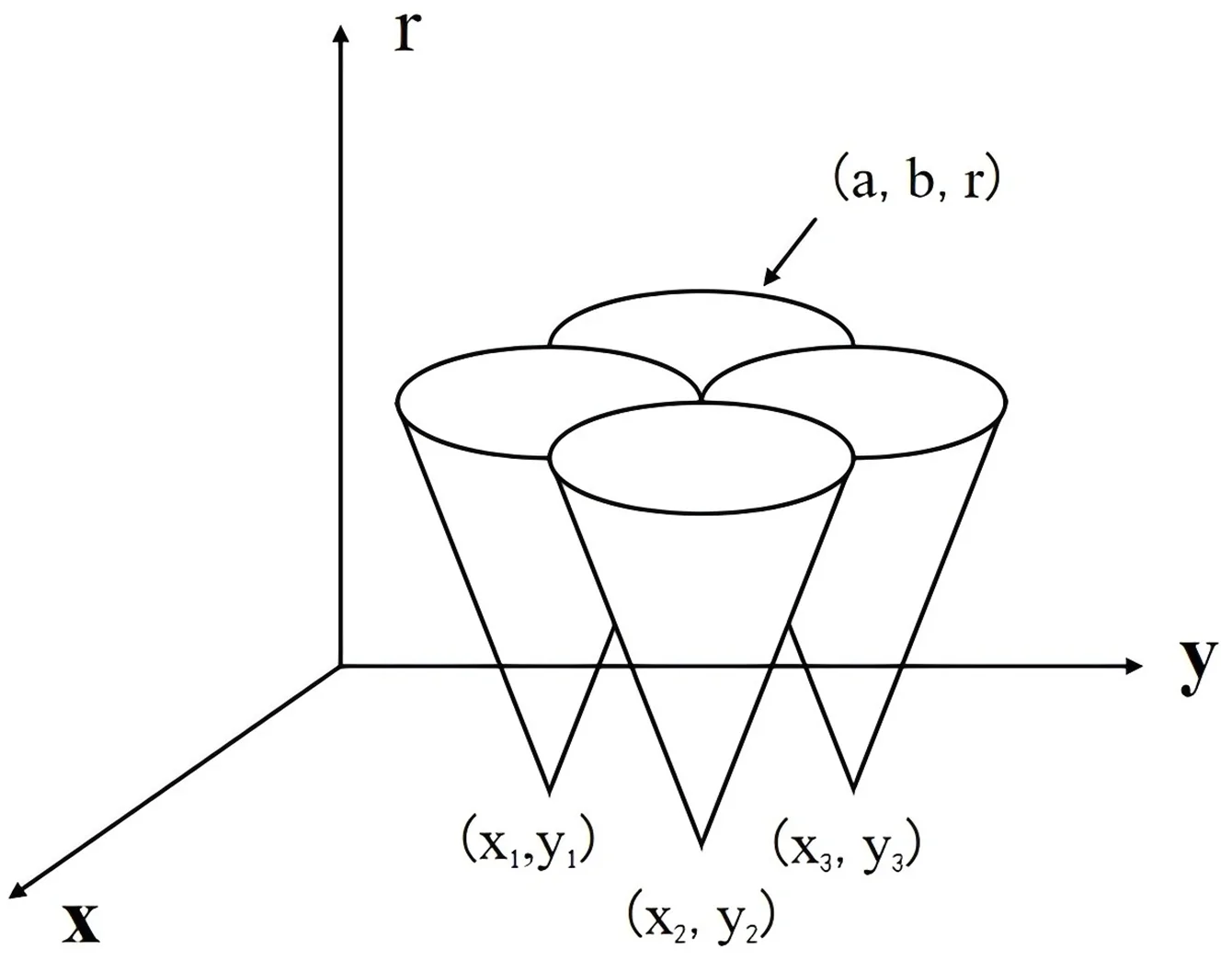
Schematic diagram of the Hough transform principle.

To ensure detection accuracy, our method applies a cascade of post-processing filters following initial circle detection. First, area-based filtering retains only disk-shaped regions with pixel areas in the range [100, 1000] px². Second, circularity filtering enforces the standard metric C = 4π·A/P² ≥ 0.8, where A denotes the region’s area and P its perimeter—thereby selecting only compact, near-circular structures. All thresholds were empirically calibrated using representative froth image samples to suppress false positives and resolve overlapping detections. The final output comprises per-bubble geometric attributes: center coordinates (x, y), radius r, and circularity C—providing a robust foundation for quantitative foam analysis. Detection and post-processing parameters are summarized in Table 2.

**Table 2:** Parameter Settings and Objectives of Hough Circle Detection and Candidate Circle Selection.

Processing Stage	Parameter	Parameter Value	Purpose
Morphological Processing	kernel size	3 x 3	To enhance the continuity of circular boundaries
Accumulator Resolution	dp	1	To ensure the localization accuracy of the circle center
Minimum Center Distance	minDist	20 px	To avoid repeated detection of the same bubble
Canny High Threshold	param1	50	To preserve the edges of core bubbles
Accumulator Threshold	param2	30	To improve the detection rate of low-contrast bubble boundaries
Minimum Radius	minRadius	5 px	To exclude minor noise points
Maximum Radius	maxRadius	100 px	To exclude large artifacts or background structures
Area Filtering	area	100 < area < 1000	To remove overly small noise and large false positives
Circularity Filtering	circularity	circularity > 0.8	To eliminate non-circular boundaries or false detections due to bubble adhesion
Result Output	output	–	To provide data for subsequent quantitative analysis

In this study, all parameters listed in Table 2 were fixed prior to experimentation and applied uniformly across the entire test set—ensuring consistent, reproducible detection performance. No manual parameter tuning was performed on individual test images, thereby guaranteeing fair cross-sample comparison. The Hough transform parameters minRadius and maxRadius were calibrated from the pixel-size distribution of bubbles observed in a representative training subset, reflecting realistic foam morphology. minDist enforces a minimum center-to-center separation to suppress redundant detections of the same bubble; param1 (Canny high threshold) governs strong-edge retention, while param2 (accumulator threshold) modulates voting sensitivity—jointly balancing detection completeness and specificity. Finally, area and circularity filtering impose geometric constraints grounded in physical plausibility: only regions with areas between 100–1000 px² and circularity ≥ 0.8 are retained, yielding robust, interpretable statistics on bubble count and size distribution.

## 4. Experiment and Evaluation

### 4.1 Experimental Environment

The experimental environment is systematically characterized across three dimensions: hardware configuration, software configuration, and model training parameters. For hardware, the platform comprises an Intel Core i7-12700K CPU and an NVIDIA RTX 3090 GPU with 24 GB of GPU memory and 32 GB of system RAM—sufficient to support U-Net model training, image segmentation inference, and subsequent Hough circle detection without computational bottlenecks.

For software, the system operates on Windows 10 (64-bit), implemented in Python 3.8 with PyTorch 1.10.0 as the deep learning backend. Image processing and post-processing leverage OpenCV 4.5.5 and PIL 9.0.1: OpenCV performs image preprocessing, Hough circle detection, and result refinement, whereas PIL handles image loading, geometric resizing, and lossless saving.

Regarding model training, the foam image dataset contains 300 valid frames acquired exclusively from the stable region of a single flotation cell. To mitigate data scarcity—a constraint imposed by industrial acquisition costs—we applied geometric augmentations (rotation and horizontal flipping) as detailed in Section 3.1, thereby enriching dataset diversity and strengthening model generalization while suppressing overfitting. The dataset spans diverse operational regimes characterized by varying foam size and density, ensuring broad representativeness. All input images are uniformly resized to 256 × 256 pixels and partitioned randomly into training, validation, and test subsets at a 7:2:1 ratio. Training employs a batch size of 8, 100 epochs, the Adam optimizer, a learning rate of 0.001, and BCELoss as the objective function. The U-Net output layer applies the Sigmoid activation function to produce pixel-wise probability maps, with dropout regularization set to 0.3. Model weights are checkpointed every 50 iterations. In the downstream feature extraction stage, binary segmentation masks generated by U-Net serve as the sole input to Hough circle detection, with all Hough parameters held invariant to guarantee reproducible, robust bubble detection performance.

### 4.2 Impact on Segmentation Performance

#### 4.2.1 Pixel-level segmentation evaluation.

The features of foam images based on U-Net and Hough transform are evaluated using the following three formulas. *TP*: the number of pixels where the true label is foam and the predicted result is also foam; *TN*: the number of pixels where the true label is background and the predicted result is also background; *FP*: the number of pixels where the true label is background but the predicted result is misclassified as foam; *FN*: the number of pixels where the true label is foam but the predicted result is misclassified as background.

(1) Pixel Accuracy(*PA*)

$$\begin{array}{c} PA=\frac{TP+TN}{TP+TN+FP+FN}\times \text{1}\text{0}\text{0}\% \end{array}$$ (8)

*PA* reflects the overall accuracy of pixel classification but is not sensitive enough to class imbalance scenarios.

(2) Intersection over Union(*IoU)*

$$\begin{array}{c} IoU=\frac{TP}{TP+FP+FN}\times \text{1}\text{0}\text{0}\% \end{array}$$ (9)

*IoU* is the core metric of semantic segmentation, directly reflecting the overlapping accuracy of the foam area. The higher the *IoU* value, the more precise the segmentation of the foam contours.

(3) Dice Similarity Coefficient(*Dice*)

$$\begin{array}{c} Dice=\frac{\text{2}\times TP}{\text{2}\times TP+FP+FN}\times \text{1}\text{0}\text{0}\% \end{array}$$ (10)

*Dice* is highly correlated with *IoU* but is more sensitive to the segmentation performance of small-sized objects. It is commonly used for accuracy assessment in scenarios such as medical imaging and industrial small target segmentation.

#### 4.2.2 Boundary quality assessment.

(1) *Boundary F1-score*

$$\begin{array}{c} BF=\frac{\text{2}\times P_{b}\times R_{b}}{P_{b}+R_{b}} \end{array}$$ (11)

$P_{\text{b}}$ : boundary precision, $R_{\text{b}}$: boundary recall. *Boundary F1-score* is used to measure the matching degree between the predicted bubble boundary and the manually labeled boundary. Different from *Dice* and *IoU*, *Boundary F1* focuses more on foam adhesion area, weak boundary area and edge positioning accuracy, so it is more suitable for evaluating the boundary segmentation quality of flotation foam image.

(2) *Boundary IoU*

$$\begin{array}{c} BI=\frac{\left|B_{p}⋂B_{g}\right|}{\left|B_{p}⋃B_{g}\right|} \end{array}$$ (12)

$B_{p}$: Predict the boundary area;$B_{g}$: Real boundary area. Ordinary *IoU* calculates the overlap of the whole region, while *Boundary IoU* only calculates the intersection ratio in the area near the boundary, so it is more sensitive to boundary offset, boundary fracture and inaccurate segmentation of adhesive foam.

In summary, this paper comprehensively evaluates the segmentation effect of foam images from the aspects of pixel-level classification accuracy, target area overlap, small target segmentation sensitivity and boundary positioning quality. Among them, PA is used to measure the correctness of the overall pixel classification. *IoU* and *Dice* are used to reflect the degree of overlap between the prediction results and the manually labeled regions. *Boundary F1-score* and *Boundary IoU* further examine the model ‘s ability to identify foam adhesion boundaries, weak boundaries and edge details. Through the joint evaluation of the above indicators, the accuracy and stability of the model in the flotation foam image segmentation task can be more comprehensively reflected. The model training results are shown in Fig 9.

**Fig 9:**
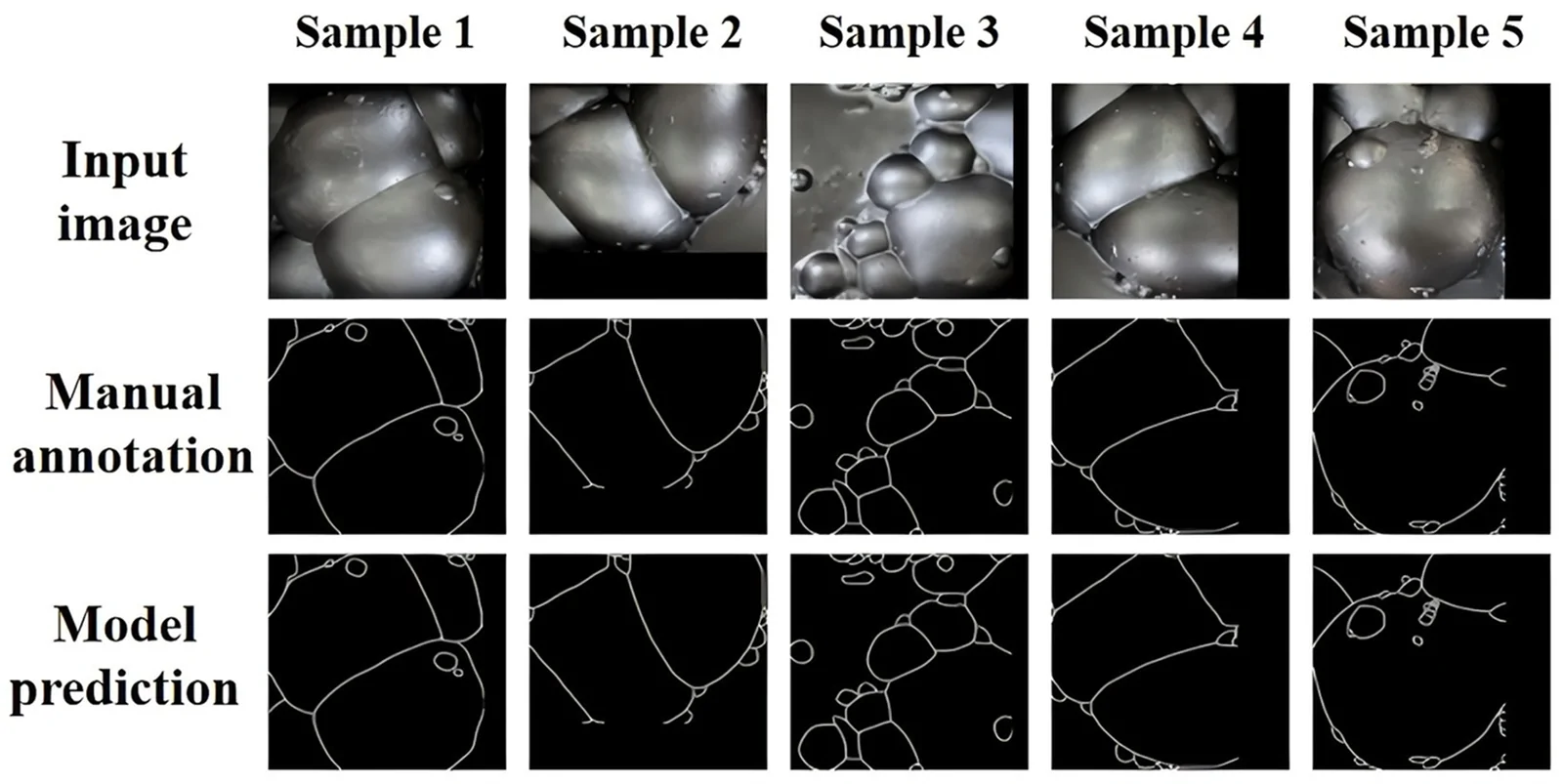
Schematic diagram of model training results.

Fig 9 offers a qualitative view of the trained model’s segmentation performance on a representative test image. The first row shows the original foam image; the second row displays the manually annotated bubble masks; and the third row presents the edge map predicted by the proposed U-Net.

By inspection, the predicted contours closely match the ground-truth annotations. Even in regions with touching and overlapping bubbles, the model delineates boundaries robustly, indicating that the network effectively translates learned representations into precise spatial delineations. This visual agreement is consistent with the quantitative results reported later.

To enable explicit visual comparison, Fig 10 presents a composite visualization for direct pixel-level evaluation of prediction against ground truth.

**Fig 10:**
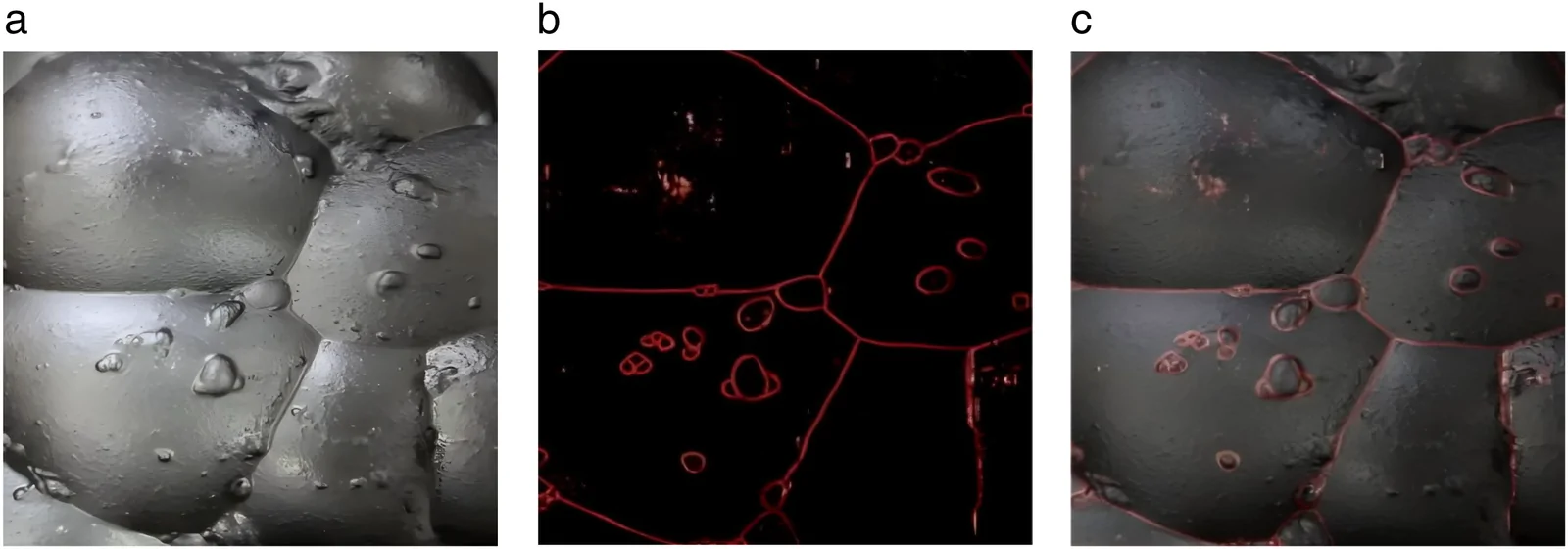
Schematic diagram of segmentation results. (a) Original image, (b) Segmented image (c) Overlapping image.

In Fig 10(a), the input foam image is shown. Fig 10(b) displays the binarized U-Net prediction as a binary mask, with foam regions in white and the background in black. Fig 10(c) superimposes the predicted boundaries onto the ground-truth boundaries; where alignment is perfect, the two are indistinguishable, enabling immediate identification of localization offsets or segmentation mismatches.

As shown in the superimposed image in Fig 10(c), the predicted boundaries exhibit strong spatial alignment with the manually annotated ground truth. The extracted contours are topologically continuous and sharply delineated, and the segmented regions faithfully preserve the characteristic elliptical or near-circular morphology of individual flotation bubbles. Importantly, the model demonstrates robust bubble separation under severe occlusion—particularly in densely packed or low-contrast regions—where conventional watershed- and threshold-based segmentation methods routinely yield fragmented or merged contours. This capability is essential for reliable Hough-circle detection, as accurate parameter estimation depends critically on well-isolated, morphologically intact bubble boundaries. Overall, the strong visual agreement in Fig 10 corroborates the quantitative results in Table 3 and validates the proposed approach for static feature extraction from flotation foam images.

**Table 3:** Comparison of segmentation performance of different preprocessing schemes.

Preprocessing Scheme	*PA* (%)	*IoU* (%)	*Dice* (%)	*Precision* (%)	*F1* (%)
NP	98.52 ± 0.08	93.04 ± 0.13	96.39 ± 0.07	96.50 ± 0.11	96.30 ± 0.10
GO	98.65 ± 0.09	93.25 ± 0.15	96.52 ± 0.08	96.65 ± 0.12	96.42 ± 0.11
BO	95.30 ± 0.18	85.70 ± 0.21	92.14 ± 0.15	92.30 ± 0.18	91.98 ± 0.16
GB	96.82 ± 0.15	89.02 ± 0.18	94.12 ± 0.12	94.25 ± 0.15	93.99 ± 0.14

Among them: NP—no preprocessing; GO—Gaussian smoothing only; BO—binarization only; GB—Gaussian smoothing followed by binarization.

Table 3 summarizes the segmentation performance under different preprocessing strategies. With no preprocessing applied, the proposed model achieves a pixel accuracy of 98.52%, an *IoU* of 93.04%, and a *Dice* coefficient of 96.39%, confirming its inherent capability for discriminative feature learning.

Gaussian smoothing marginally enhances segmentation robustness by attenuating high-frequency noise. In contrast, binarization substantially degrades performance, as it discards grayscale variations and textural details within foam regions—consequently compromising critical boundary information.

It can be seen from Table 4 that the proposed model achieves high segmentation accuracy without preprocessing. Specifically, *PA*, *IoU*, and *Dice* reach 96.52%, 93.04%, and 96.39%, respectively—demonstrating accurate pixel-level classification of foam regions versus background and strong spatial agreement with expert annotations. Precision and Recall stand at 96.50% and 96.30%, respectively, reflecting a balanced trade-off between false positives and false negatives, and consistent identification of foam boundaries.

**Table 4:** Statistics of overall segmentation performance of the model.

Evaluation Indicator	No preprocessing scheme	Inference Time(ms)
*PA*(%)	98.52 ± 0.08	16.45
*IoU*(%)	93.04 ± 0.13	18.37
*Dice*(%)	96.39 ± 0.07	16.29
*Precision*(%)	96.50 ± 0.11	17.31
*F1*(%)	96.30 ± 0.10	17.22
*Boundary F1-score*(%)	94.82 ± 0.015	18.42
*Boundary IoU*(%)	91.15 ± 0.092	17.48

Moreover, the *boundary F1* score and *boundary IoU* attain 94.82% and 91.15%, respectively, confirming not only high regional segmentation accuracy but also precise localization of foam boundaries. Flotation foam images commonly exhibit challenges such as touching and overlapping bubbles, as well as low-contrast boundaries; thus, boundary quality metrics directly assess the model’s capability to resolve fine contour details. These superior boundary metrics demonstrate that the proposed model delivers reliable boundary maps for subsequent Hough circle detection—enabling robust extraction of static features including foam count, radius, diameter, perimeter, and area.

To further evaluate the effectiveness of the proposed method, we compare it against five representative semantic segmentation models: FCN-8s [29], M_S_J [30], TransFuse [31], DeepLabv3 and GC-FSegNet [32].

It can be seen from Table 5 that the proposed U-Net achieves superior performance in both *PA* and *Dice*. Specifically, its *PA* reaches 96.52%, outperforming FCN-8s [29], M_S_J [30], TransFuse [31], DeepLabv3 and GC-FSegNet [32]—demonstrating superior pixel-level classification accuracy for foam regions versus background and a marked reduction in misclassification errors.

**Table 5:** Test results and performance indexes of different models.

algorithm	*PA*(%)	*Dice*(%)
FCN-8_S_ [29]	84.78	90.80
M_S_J [30]	90.95	94.78
Trans Fuse [31]	90.77	95.14
DeepLabv3 [32]	84.90	90.82
GC-FSegNet [32]	85.82	94.43
U-Net	96.52	96.39

Regarding the *Dice* coefficient, the proposed U-Net attains 96.39%, exceeding all comparative models and confirming stronger spatial overlap between predictions and expert annotations. Compared with FCN-8s, the encoder-decoder architecture with skip connections better preserves fine-grained spatial details along foam boundaries. In contrast to M_S_J, TransFuse, and GC-FSegNet, the proposed model maintains robust boundary recovery in challenging scenarios—including touching and overlapping bubbles, low-contrast boundaries, and complex contour configurations.

Overall, the proposed U-Net delivers high *PA* and *Dice* scores, reflecting both exceptional global classification accuracy and comprehensive foam contour structure preservation. These results demonstrate that the model generates reliable boundary maps for subsequent Hough circle detection, thereby improving the stability and accuracy of static feature extraction—including foam count, radius, diameter, perimeter, and area.

## 5. System integration and industrial application prospect

In order to verify the practicality of this study and facilitate transition to industrial applications, we developed a GUI prototype system. The platform integrates a complete processing pipeline: image input, preprocessing selection, U-Net inference, Hough detection, and result visualization.

The GUI provides an intuitive parameter adjustment panel for field operators, mainly including the following modules: Preprocessing module: Gaussian smoothing can be selected, with adjustable kernel size; or binarization can be selected, along with an adaptive thresholding algorithm. U-Net module: different pre-trained weight files can be loaded to adapt to varying flotation conditions. Hough module: this is the core of tuning—the parameters include the high and low Canny thresholds, the center accumulator threshold, and the minimum and maximum radii of detected circles. These parameters have reasonable default values, and users can fine-tune them according to the characteristics of the current foam image to optimize detection performance. The interface displays the original image, segmentation map, and detection overlay in real time, while listing attributes and statistical information for each detected bubble.

The system design follows the principles of clear workflow and simple operation. Through tab-based organization, parameter adjustments are implemented via sliders and input boxes, with all operations providing immediate visual feedback.

Currently, the prototype system has completed functional verification and stability testing in the laboratory environment, and reliably processes offline-collected foam image sequences. Although not yet deployed online in an operational flotation plant, the system design fully anticipates industrial requirements—such as modular architecture for integration into existing monitoring systems and on-device computation to ensure data security. The next step is to collaborate with industrial partners on pilot deployment in the flotation workshop of a coal preparation plant, validating system robustness under real-world noise, illumination variations, and process fluctuations, while further optimizing algorithmic and GUI real-time performance.

## 6. Conclusion

This study proposes a flotation froth image analysis framework based on a boundary-aware U-Net and a segmentation-guided adaptive Hough transform for static feature extraction. The method addresses typical challenges in froth-image analysis, including touching and overlapping bubbles, low-contrast boundaries, and variations in image quality. By introducing boundary-oriented supervision into the segmentation stage, the proposed model improves delineation of adjacent bubbles and generates more reliable structural information for subsequent geometric analysis.

On the basis of the segmentation results, an adaptive Hough-based detection stage extracts bubble-related geometric features—including bubble count, radius, diameter, perimeter, and area—thereby establishing a robust link between pixel-level semantic segmentation and quantitative feature extraction. Experimental results demonstrate that the proposed method achieves high segmentation performance, with an Intersection over Union (*IoU*) of 93.04% a *Dice* similarity coefficient of 96.39%, while maintaining favorable inference efficiency.

Compared with several representative segmentation models, the proposed method strikes a superior balance between accuracy and computational cost, confirming its suitability for practical froth-image analysis. Future work will focus on expanding the dataset to cover more diverse operating conditions, enhancing robustness against industrial disturbances (e.g., illumination shifts and mechanical vibrations), and validating the framework in online flotation monitoring systems.

The main contributions of this paper are as follows: 1) An end-to-end pipeline integrating U-Net segmentation and Hough circle detection for static feature extraction; 2) A GUI-based operating platform supporting real-time parameter adjustment, thereby facilitating industrial deployment; 3) Architecture and parameter optimization tailored to froth image characteristics, enabling stable performance under challenging conditions—including bubble touching and size heterogeneity.
